# German nation-wide in-patient treatment of abdominal aortic aneurysm—trends between 2005 and 2019 and impact of the SARS-CoV-2 pandemic

**DOI:** 10.1186/s42155-023-00389-4

**Published:** 2023-08-29

**Authors:** Stefanie Bette, Josua A. Decker, Sebastian Zerwes, Yvonne Gosslau, Dominik Liebetrau, Alexander Hyhlik-Duerr, Florian Schwarz, Thomas J. Kroencke, Christian Scheurig-Muenkler

**Affiliations:** 1 Diagnostic and Interventional Radiology, Faculty of Medicine, University Hospital Augsburg, University of Augsburg, Stenglinstr. 2, 86156 Augsburg, Germany; 2 Vascular Surgery, Faculty of Medicine, University of Augsburg, Stenglinstr. 2, 86156 Augsburg, Germany; 3 Diagnostic and Interventional Radiology, Donauisar Klinikum Deggendorf, Perlasberger Str. 41, 94469 Deggendorf, Germany; 4 Centre for Advanced Analytics and Predictive Sciences (CAAPS), University of Augsburg, Universitätsstr. 2, 86159 Augsburg, Germany

**Keywords:** COVID-19 pandemic and AAA, Abdominal aortic aneurysm, Ruptured abdominal aortic aneurysm, Epidemiological changes in AAA treatment, EVAR

## Abstract

**Purpose:**

Aim of this study was to analyze hospitalizations due to ruptured and non-ruptured abdominal aortic aneurysms (rAAA, nrAAA) in Germany between 2005 and 2021 to determine long-term trends in treatment and the impact of the SARS-CoV-2 pandemic.

**Materials and Methods:**

Fully anonymized data were available from the research data center (RDC) of the German Federal Statistical Office (Destatis). All German hospitalizations with the ICD-10 code “I71.3, rAAA” and “I71.4, nrAAA” in 2005 and 2010–2021 were analyzed.

**Results:**

We report data of a total of 202,951 hospitalizations. The number of hospitalizations increased from 2005 to 2019 (14,075 to 16,051, + 14.0%). The rate of open repair (OR) constantly decreased, whereas the rate of endovascular aortic repair (EVAR) increased until 2019. During the pandemic, the number of hospitalizations due to nrAAA dropped from 13,887 (86.5%) in 2019 to 11,278 (85.0%) in 2021. The strongest decrease of hospitalizations for AAA was observed during the first wave of the SARS-CoV-2-pandemic in spring 2020 (-25.5%).

**Conclusion:**

Over the past decades, we observed an increasing number of hospitalizations due to AAA accompanied by a shift from OR to EVAR especially for nrAAA. During the lockdown measures due to the SARS-CoV-2-pandemic, a decrease in hospitalizations for nrAAA (but not for rAAA) was shown in 2020 and furthermore in 2021 with no rebound of treatment of nrAAA suggesting an accumulation of untreated AAA with a potentially increased risk of rupture.

**Supplementary Information:**

The online version contains supplementary material available at 10.1186/s42155-023-00389-4.

## Introduction

Abdominal aortic aneurysms (AAA) are a potentially life-threatening disease with a prevalence of approximately 4% in (mainly male) patients over 65 years of age [1]. AAA are mostly asymptomatic, mortality of patients with ruptured AAA (rAAA) is estimated to be about 60–80% [2–4]. Therefore, early detection of AAA is important and screening methods have been introduced in recent years [3, 5]. During the past decade, epidemiology and treatment regimens of AAA have changed; outcome improved predominantly due to early detection of AAA in screening programs and advances in treatment, e.g. the introduction of endovascular aortic repair (EVAR). According to a previous study, the incidence of non-ruptured AAA (nrAAA) in Germany has increased in recent years, while the incidence of rAAA decreased until 2014 [2].

Nationwide healthcare data of in-hospital treatment—as provided by The German Federal Statistical Office (Destatis)—give opportunity for detailed analysis of relevant long-term trends but also allows to study the impact of exceptional conditions such as the SARS-CoV-2 pandemic on the German healthcare system.

Thus, the aim of this study was to analyze long-term trends of hospitalizations and treatment regimens for rAAA and nrAAA in Germany from 2005 to 2019 and to identify the effects of the SARS-CoV-2 pandemic in 2020 and 2021.

## Materials & methods

### Data collection, patient cohort, diagnoses, and procedures

The detailed procedure of data acquisition has been reported in detail previously [6]. In brief, the research data center (RDC) of the German Federal Statistical Office (Destatis) provided fully anonymized data after receiving syntaxes written by the authors in Stata 17 (www.stata.com) [7]. No approval of the Medical Research and Ethics Committee (MREC) or informed consent were necessary due to the analysis of fully anonymized data.

According to the International Classification of Diseases, Tenth Revision (ICD-10) the following diagnoses were analyzed in the years 2005 and 2010–2021: ruptured abdominal aortic aneurysm (ICD-10 code: I71.3) and non-ruptured abdominal aortic aneurysm (I71.4). The following secondary diagnoses were evaluated: primary hypertension (I10), diabetes mellitus type 2 (E11), diseases of lipoprotein metabolism (E78), chronic kidney disease (N18), ischemic heart disease (I25) and SARS-CoV-2 disease (U07.1).

Using specific Operating and Procedure (OPS) codes the following treatment regimens were categorized: Surgical treatment (open repair [OR]) and endovascular aortic repair [EVAR]). Specific OPS-codes are listed in Supplemental Table 1. No treatment was defined as absence of all defined OPS-codes, a combined endovascular and surgical treatment was defined as presence of at least one OPS-code from the group OR and EVAR.

For all hospitalizations with rAAA and nrAAA and for the defined subgroups the following parameters were recorded: gender, age, duration of in-hospital stay, and in-hospital mortality.

Further, semimonthly (01.-14. and 15.-end of month) data for all hospitalizations were analyzed. In 2020 and 2021, the impact of the four waves of the SARS-CoV-2 pandemic was analyzed. Waves of infections were defined according to previously published data from the Robert-Koch-Institute Germany [8].

### Statistical analyses

Data are shown as absolute numbers (n) and percentages (%), as mean (± sd) or as median (IQR). Absolute and relative changes between 2005 and 2019, between 2019 and 2020 and between 2020 and 2021 as well as in pre-defined time periods were calculated. All statistical analyses were performed using SPSS 28.0 (IBM statistics).

## Results

**Fig. 1:**
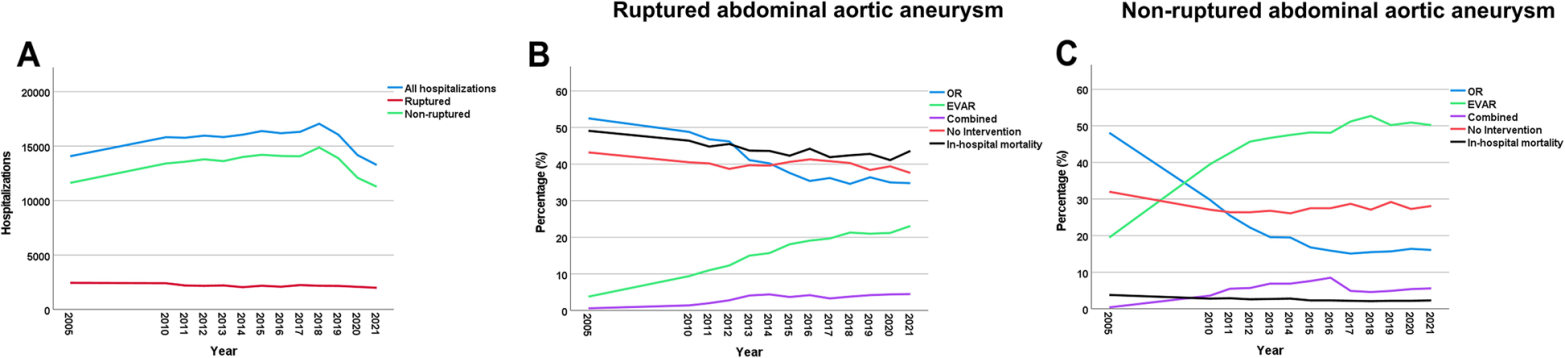
Hospitalizations due to ruptured and non-ruptured abdominal aortic aneurysm (AAA) between 2005 and 2021 (**A**). Trends of different treatment regimens and in-hospital mortality for ruptured (**B**) and non-ruptured (**C**) AAA between 2005 and 2021. (OR: open repair, EVAR: endovascular aortic repair)

A total of 202,951 hospitalizations (male: 172,752 [85.1%], mean age 73.2 years) with the diagnoses rAAA or nrAAA between 2005 and 2021 were analyzed. The number of hospitalizations increased by 14.0% from 2005 (*n* = 14,075) to 2019 (*n* = 16,051). The highest increase was observed in the year 2018 (*n* = 17,056). In 2020, the number decreased to *n* = 14,178 (change -1,873, -11.7%). Detailed information on demographic data, comorbidities treatment and outcome are given in Tables 1 and 2 and Fig. 1. Data for all hospitalizations (years 2005, 2010–2019) are summarized in Supplemental Table 2.

**Table 1:** Hospitalizations due to ruptured and non-ruptured abdominal aortic aneurysm between 2005 and 2019

Year	2005	2010	2015	2019	Absolute Change 2005—2019	Rel. Change 2005—2019
**All hospitalizations, n**	14,075	15,819	16,385	16,051	+ 1,976	** + 14.0%**
Age, mean (± sd)	71.94 (± 8.63)	72.79 (± 8.83)	73.25 (± 9.06)	73.81 (± 9.04)	+ 1.87	** + 2.6%**
Men, n (%)	12,058 (85.7%)	13,621 (86.1%)	13,864 (84.6%)	13,663 (85.1%)	+ 1,605 (-0.5%)	**-0.6%**
In-hospital death, n (%)	1,642 (11.7%)	1,497 (9.5%)	1,252 (7.6%)	1,235 (7.7%)	-407 (-4.0%)	**-34.0%**
In-hospital stay, d, median (IQR)	11 (4–16)	9 (5–14)	7 (4–12)	7 (4–11)	-4	**-36.4%**
**Comorbidities:**						
Primary HT	7,900 (56.1%)	9,853 (62.3%)	10,715 (65.4%)	10,775 (67.1%)	+ 2,875 (+ 11.0%)	** + 19.6%**
DLP	3,140 (22.3%)	4,929 (31.2%)	6,115 (37.3%)	6,553 (40.8%)	+ 3,413 (+ 18.5%)	** + 83.0%**
DM-2	1,619 (11.5%)	2,669 (16.9%)	2,963 (18.1%)	2,731 (17.0%)	+ 1,112 (+ 5.5%)	** + 47.9%**
CKD	2,212 (15.7%)	3,491 (22.1%)	3,948 (24.1%)	3,861 (24.1%)	+ 1,649 (+ 8.3%)	** + 53.1%**
CHD	4,967 (35.3%)	5,248 (33.2%)	5,295 (32.3%)	5,060 (31.5%)	+ 93 (-3.8%)	**-10.7%**
**Ruptured abdominal aortic aneurysm**						
Hospitalizations, n (%)	2,449 (17.4%)	2,410 (15.2%)	2,180 (13.3%)	2,164 (13.5%)	-285 (-3.9%)	**-22.5%**
Age, mean (± sd)	74.77 (± 9.64)	75.16 (± 10.19)	75.70 (± 10.23)	76.10 (± 10.11)	+ 1.33	** + 1.8%**
Men, n (%)	1,942 (79.3%)	1,948 (80.8%)	1,723 (79.0%)	1,743 (80.5%)	-199 (+ 1.2%)	** + 1.6%**
**Treatment:**						
OR, n (%)	1,286 (52.5%)	1,176 (48.8%)	820 (37.6%)	788 (36.4%)	-498 (-16.1%)	**-30.7%**
EVAR, n (%)	92 (3.8%)	226 (9.4%)	395 (18.1%)	455 (21.0%)	+ 363 (+ 17.3%)	** + 459.7%**
combined, n (%)	14 (0.6%)	33 (1.4%)	81 (3.7%)	90 (4.2%)	+ 76 (+ 3.6%)	** + 627.5%**
no intervention, n (%)	1,057 (43.2%)	975 (40.5%)	884 (40.6%)	831 (38.4%)	-226 (-4.8%)	**-11.0%**
**In-hospital death, n (%)**	1,202 (49.1%)	1,118 (46.4%)	922 (42.3%)	927 (42.8%)	-275 (-6.2%)	**-12.7%**
**In-hospital stay, d, median (IQR)**	5 (1–18)	5 (1–17)	6 (1–17)	6 (1–16)	+ 1	** + 20.0%**
**Non-ruptured abdominal aortic aneurysm**						
Hospitalizations, n	11,626 (82.6%)	13,409 (84.8%)	14,205 (86.7%)	13,887 (86.5%)	+ 2,261 (+ 3.9%)	**4.7%**
Age, mean (± sd)	71.35 (± 8.28)	72.36 (± 8.49)	72.87 (± 8.81)	73.45 (± 8.81)	+ 2.10	** + 2.9%**
Men, n (%)	10,116 (87.0%)	11,673 (87.1%)	12,141 (85.5%)	11,920 (85.8%)	+ 1,804 (-1.2%)	**-1.4%**
**Treatment:**						
OR, n (%)	5,588 (48.1%)	3,997 (29.8%)	2,385 (16.8%)	2,176 (15.7%)	-3,412 (-32.4%)	**-67.4%**
EVAR, n (%)	2,264 (19.5%)	5,293 (39.5%)	6,840 (48.2%)	6,970 (50.2%)	+ 4,706 (+ 30.7%)	** + 157.7%**
combined, n (%)	50 (0.4%)	481 (3.6%)	1,079 (7.6%)	678 (4.9%)	+ 637 (+ 4.5%)	** + 1,050.3%**
no intervention, n (%)	3,724 (32.0%)	3,638 (27.1%)	3,901 (27.5%)	4,054 (29.2%)	+ 330 (-2.8%)	**-8.9%**
In-hospital death, n (%)	440 (3.8%)	379 (2.8%)	330 (2.3%)	308 (2.2%)	-132 (-1.6%)	**-41.4%**
In-hospital stay, d, median (IQR)	12 (6–16)	9 (6–14)	7 (5–12)	7 (4–10)	-5	**-41.7%**

*OR* Open repair, *EVAR* Endovascular aneurysm repair, *Sd* Standard deviation, *IQR* Interquartile range, *HT* Hypertension, *DLP* Diseases of lipoprotein metabolism, *DM-2* Diabetes mellitus type 2, *CKD* Chronic kidney disease, *CHD* Chronic heart disease

**Table 2:** Hospitalizations due to ruptured and non-ruptured aortic aneurysm in 2019, 2020 and 2021

	**2019**	**2020**	**2021**	**Absolute Change**	**Rel. Change**	**Absolute Change**	**Rel. Change**
				**2019–2020**	**2019–2020**	**2020–2021**	**2020–2021**
**All hospitalizations, n**	16,051	14,178	13,273	-1,873	**-11.7%**	-905	**-6.4%**
Age, mean (± sd)	73.81 (± 9.04)	73.46 (± 9.14)	73.41 (± 9.23)	-0.35	**-0.5%**	-0.05	**-0.1%**
Men, n (%)	13,663 (85.1%)	12,039 (84.9%)	11,150 (84.0%)	-1,624 (-0.2%)	**-0.2%**	-889 (-0.9%)	**-1.1%**
In-hospital death, n (%)	1,235 (7.7%)	1,117 (7.9%)	1,129 (8.5%)	-118 (+ 0.2%)	**2.4%**	+ 12 (+ 0.6%)	**8.0%**
In-hospital stay, d, median (IQR)	7 (4–11)	6 (4–11)	6 (4–10)	-1	**-14.3%**	0	**0.0%**
**Comorbidities:**							
Primary HT	10,775 (67.1%)	9,782 (69.0%)	9,146 (68.9%)	-993 (+ 1.9%)	**2.8%**	-636 (-0.1%)	**-0.1%**
DLP	6,553 (40.8%)	5,858 (41.3%)	5,711 (43.0%)	-695 (+ 0.5%)	**1.2%**	-147 (+ 1.7%)	**4.1%**
DM-2	2,731 (17.0%)	2,327 (16.4%)	2,279 (17.2%)	-404 (-0.6%)	**-3.5%**	-48 (+ 0.8%)	**4.6%**
CKD	3,861 (24.1%)	3,158 (22.3%)	2,946 (22.2%)	-703 (-1.8%)	**-7.4%**	-212 (-0.1%)	**-0.4%**
CHD	5,060 (31.5%)	4,302 (30.3%)	4,100 (30.9%)	-758 (-1.2%)	**-3.7%**	-202 (+ 0.5%)	**1.8%**
SARS-CoV2-infection	0 (0.0%)	49 (0.3%)	64 (0.5%)	+ 49 (+ 0.3%)	**n/a**	+ 15 (+ 0.1%)	**39.5%**
**Ruptured aortic abdominal aneurysm**							
Hospitalizations, n	2,164 (13.5%)	2,080 (14.7%)	1,995 (15.0%)	-84 (+ 1.2%)	**8.8%**	-85 (+ 0.4%)	**2.5%**
Age, mean (± sd)	76.10 (± 10.11)	75.53 (± 10.36)	75.29 (± 10.43)	-0.58	**-0.8%**	-0.24	**-0.3%**
Men, n (%)	1,743 (80.5%)	1,639 (78.8%)	1,585 (79.4%)	-104 (-1.7%)	**-2.2%**	-54 (+ 0.7%)	**0.8%**
**Treatment:**							
OR, n (%)	788 (36.4%)	728 (35.0%)	695 (34.8%)	-60 (-1.4%)	**-3.9%**	-33 (-0.2%)	**-0.5%**
EVAR, n (%)	455 (21.0%)	440 (21.2%)	461 (23.1%)	-15 (+ 0.1%)	**0.6%**	+ 21 (+ 2.0%)	**9.2%**
combined, n (%)	90 (4.2%)	92 (4.4%)	89 (4.5%)	+ 2 (+ 0.3%)	**6.4%**	-3 (0.0%)	**0.9%**
no intervention, n (%)	831 (38.4%)	820 (39.4%)	750 (37.6%)	-11 (+ 1.0%)	**2.7%**	-70 (-1.8%)	**-4.6%**
In-hospital death, n (%)	927 (42.8%)	854 (41.1%)	870 (43.6%)	-73 (-1.8%)	**-4.2%**	+ 16 (+ 2.6%)	**6.2%**
In-hospital stay, d, median (IQR)	6 (1–16)	6.5 (1–17)	6 (1–16)	0.5	**8.3%**	-0.5	**-7.7%**
**Non-ruptured abdominal aortic aneurysm**							
Hospitalizations, n	13,887 (86.5%)	12,098 (85.3%)	11,278 (85.0%)	-1,789 (-1.2%)	**-1.4%**	-820 (-0.4%)	**-0.4%**
Age, mean (± sd)	73.45 (± 8.81)	73.11 (± 8.87)	73.08 (± 8.96)	-0.34	**-0.5%**	-0.03	**0.0%**
Men, n (%)	11,920 (85.8%)	10,400 (86.0%)	9,565 (84.8%)	-1,520 (+ 0.1%)	**0.2%**	-835 (-1.2%)	**-1.3%**
**Treatment:**							
OR, n (%)	2,176 (15.7%)	1,984 (16.4%)	1,816 (16.1%)	-192 (+ 0.7%)	**4.7%**	-168 (-0.3%)	**-1.8%**
EVAR, n (%)	6,970 (50.2%)	6,159 (50.9%)	5,660 (50.2%)	-811 (+ 0.7%)	**1.4%**	-499 (-0.7%)	**-1.4%**
combined, n (%)	678 (4.9%)	658 (5.4%)	636 (5.6%)	-29 (+ 0.5%)	**9.9%**	-22 (+ 0.2%)	**3.7%**
no intervention, n (%)	4,054 (29.2%)	3,297 (27.3%)	3,166 (28.1%)	-757 (-1.9%)	**-6.6%**	-131 (+ 0.8%)	**3.0%**
In-hospital death, n (%)	308 (2.2%)	263 (2.2%)	259 (2.3%)	-45 (-0.04%)	**-2.0%**	-4 (+ 0.1%)	**5.6%**
In-hospital stay, d, median (IQR)	7 (4–10)	6 (4–10)	6 (4–10)	-1	**-14.3%**	0	**0.0%**

*OR* Open repair, *EVAR* Endovascular aneurysm repair, *Sd* Standard deviation, *IQR* Interquartile range, *HT* Hypertension, *DLP* Diseases of lipoprotein metabolism, *DM-2* Diabetes mellitus type 2, *CKD* Chronic kidney disease, *CHD* Chronic heart disease

During the SARS-CoV2-pandemic we observed a decrease in hospitalizations: in 2020, a total of 14,178 hospitalizations (rel. decrease compared to 2019: 11.7%) were reported, in 2021 a further decrease was shown (*n* = 13,273 in 2021, rel. decrease compared to 2020: 6.4%).

In-hospital mortality increased during the pandemic from previous 7.7% in 2019 to 7.9% in 2020 and 8.5% in 2021.

**Fig. 2:**
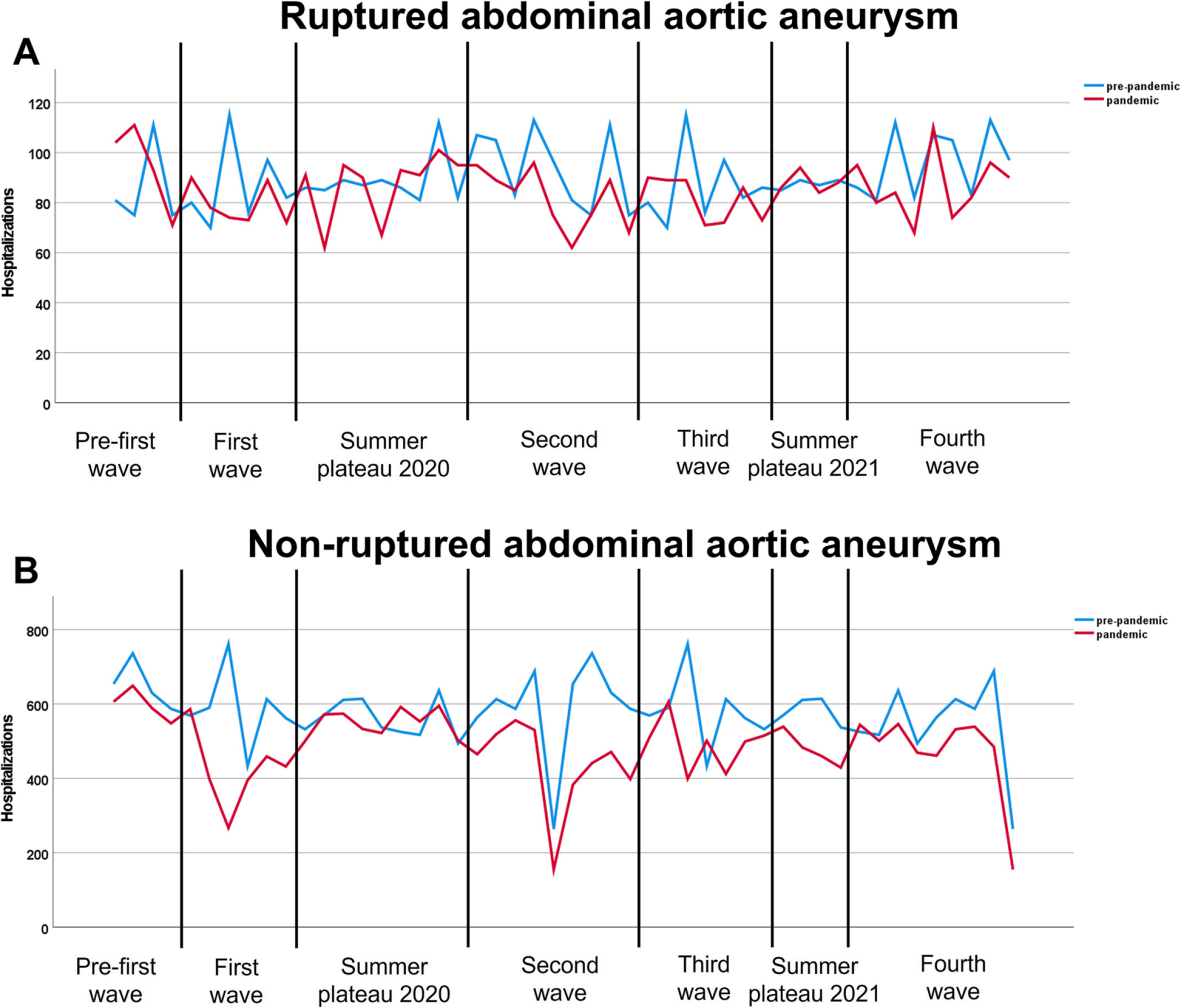
Hospitalizations due to ruptured (**A**) and non-ruptured abdominal aortic aneurysm (**B**) during the SARS-CoV2-pandemic. Corresponding hospitalization numbers from 2019 serve as pre-pandemic reference. The pandemic waves as well as the periods in between are defined according to data published by the Robert-Koch Institute [8] – first wave: March to May 2020, second wave: October 2020 to February 2021, third wave: March to May 2021, fourth wave: August to December 2021. The time frames before and between these periods were defined as pre-first wave as well as summer plateaus 2020 and 2021

During the SARS-CoV2-pandemic, a relative decrease of 25.5% was shown during the first wave of infection, followed by stable numbers during the summer plateau 2020 (-1.8%), a decrease during the second (-22.4%), third (-14.0%) and fourth (-12.9%) wave of infection as well as a decrease during the summer plateau 2021 (-15.6%) (Table 3, Fig. 2).

**Table 3:** Hospitalizations due to ruptured and non-ruptured abdominal aortic aneurysm during the SARS-CoV2- pandemic in 2020 and 2021 compared to a corresponding pre-pandemic period

	**Pre-pandemic**	**Pandemic**	**Absolute change, n (%)**	**Rel. change**
**All hospitalizations**				
Pre-first wave (Jan-Feb 2020)	2,949	2,771	-178	**-6.0%**
First wave (March–May 2020)	4,048	3,014	-1,034	**-25.5%**
Summer plateau 2020 (June-Sep 2020)	5,833	5,728	-105	**-1.8%**
Second wave (Oct 2020- Feb 2021)	5,992	4,652	-1,340	**-22.4%**
Third wave (March–May 2021)	4,666	4,012	-654	**-14.0%**
Summer plateau 2021 (June-July 2021)	2,682	2,263	-419	**-15.6%**
Fourth wave (August-Dec 2021)	5,754	5,011	-743	**-12.9%**
**Ruptured – all**				
Pre-first wave (Jan-Feb 2020)	342 (11.6%)	379 (13.7%)	+ 37 (+ 2.1%)	**17.9%**
First wave (March–May 2020)	520 (12.8%)	476 (15.8%)	-44 (+ 2.9%)	**22.9%**
Summer plateau 2020 (June-Sep 2020)	797 (13.7%)	785 (13.7%)	-12 (0.0%)	**0.3%**
Second wave (Oct 2020- Feb 2021)	884 (14.8%)	734 (15.8%)	-150 (+ 1.0%)	**6.9%**
Third wave (March–May 2021)	606 (13.0%)	570 (14.2%)	-36 (+ 1.2%)	**9.4%**
Summer plateau 2021 (June-July 2021)	350 (13.0%)	352 (15.6%)	+ 2 (+ 2.5%)	**19.2%**
Fourth wave (August-Dec 2021)	866 (15.1%)	779 (15.5%)	-87 (+ 0.5%)	**3.3%**
**Non-ruptured—all**				
Pre-first wave (Jan-Feb 2020)	2,607 (88.4%)	2,392 (86.3%)	-215 (-2.1%)	**-2.4%**
First wave (March–May 2020)	3,528 (87.2%)	2,538 (84.2%)	-990 (-2.9%)	**-3.4%**
Summer plateau 2020 (June-Sep 2020)	5,036 (86.3%)	4,943 (86.3%)	-93 (0.0%)	**0.0%**
Second wave (Oct 2020- Feb 2021)	5,108 (85.2%)	3,918 (84.2%)	-1,190 (-1.0%)	**-1.2%**
Third wave (March–May 2021)	4,060 (87.0%)	3,442 (85.9%)	-618 (-1.2%)	**-1.4%**
Summer plateau 2021 (June-July 2021)	2,332 (87.0%)	1,911 (84.4%)	-421 (-2.5%)	**-2.9%**
Fourth wave (August-Dec 2021)	4,888 (84.9%)	4,232 (84.5%)	-656 (-0.5%)	**-0.6%**
**Non-ruptured – OR**				
Pre-first wave (Jan-Feb 2020)	419 (16.1%)	413 (17.3%)	-6 (+ 1.2%)	**7.4%**
First wave (March–May 2020)	567 (16.1%)	395 (15.6%)	-172 (-0.5%)	**-3.2%**
Summer plateau 2020 (June-Sep 2020)	759 (15.1%)	804 (16.3%)	+ 45 (+ 1.2%)	**7.9%**
Second wave (Oct 2020- Feb 2021)	844 (16.5%)	675 (17.2%)	-169 (+ 0.7%)	**4.3%**
Third wave (March–May 2021)	641 (15.8%)	537 (15.6%)	-104 (-0.2%)	**-1.2%**
Summer plateau 2021 (June-July 2021)	362 (15.5%)	318 (16.6%)	-44 + 1.1%)	**7.2%**
Fourth wave (August-Dec 2021)	754 (15.4%)	658 (15.5%)	-96 (+ 0.1%)	**0.8%**
**Non-ruptured—EVAR**				
Pre-first wave (Jan-Feb 2020)	1,323 (50.7%)	1,193 (49.9%)	-130 (-0.9%)	**-1.7%**
First wave (March–May 2020)	1,769 (50.1%)	1,361 (53.6%)	-408 (+ 3.5%)	**6.9%**
Summer plateau 2020 (June-Sep 2020)	2,553 (50.7%)	2,472 (50.0%)	-81 (-0.7%)	**-1.4%**
Second wave (Oct 2020- Feb 2021)	2,518 (49.3%)	1,998 (51.0%)	-520 (+ 1.7%)	**3.4%**
Third wave (March–May 2021)	2,038 (50.2%)	1,772 (51.5%)	-266 (+ 1.3%)	**2.6%**
Summer plateau 2021 (June-July 2021)	1,191 (51.1%)	903 (47.3%)	-288 (-3.8%)	**-7.5%**
Fourth wave (August-Dec 2021)	n.a	n.a	n.a	**n.a**

The pre-pandemic period is defined as the corresponding time frame in 2019 / beginning of 2020 before the SARS-CoV2-pandemic started in 03/2020

*OR* Open repair, *EVAR* Endovascular aortic repair

### Ruptured abdominal aortic aneurysm

We report data of a total of 28,420 hospitalizations (male: 22,687 [79.8%], mean age 75.5 years) due to rAAA between 2005 and 2021. The absolute number and the rate of rAAA constantly decreased by 22.5% from 2005 until 2019 (2,449 to 2,164; 17.4 to 13.5%). The rate of OR decreased until 2019 (52.5% in 2005 to 36.4% in 2019, relative change -30.7%), whereas a strong increase of the rate of EVAR was observed (3.8% in 2005 to 21.0% in 2019, rel. change + 459.7%). The rate of patients receiving combined treatment increased from 0.6% to 4.2% (rel. change + 627.5%). In-hospital mortality decreased from 49.1% in 2005 to 42.8% in 2019 (-12.7%) (Table 1, Fig. 1B).

In 2020 and 2021, there was a stable absolute number of cases, but a relative increase in the rate of rAAA (+ 8.8% in 2020 compared to 2019 and + 2.5% in 2021 compared to 2020). The other trends continued in a similar manner as described above (Table 2). Especially the rate of EVAR increased in 2021 compared to 2020 (rel. change + 9.2%). Regarding semimonthly data, we observed an increase in the pre-first wave period (+ 10.8%), followed by a relative increase during the first wave (+ 22.9%), the second wave (+ 6.9%), the third wave (+ 9.4%) and the fourth wave (+ 3.3%) of the SARS-CoV-2 pandemic (Table 3, Fig. 2). We recorded a relative increase in the rate of rAAA during the summer plateau 2021 (+ 19.2%) with however stable absolute numbers. Numbers of hospitalizations for specific treatment regimens were too low to perform these analyses as many cases were censored.

### Non-ruptured abdominal aortic aneurysm

A total of 174,531 hospitalizations (150,065 males [85.9%], mean age 72.8 years) due to nrAAA were recorded. Absolute as well as relative numbers of nrAAA increased between 2005 and 2019 (11,626 [82.6%] in 2005 to 13,887 [86.5%] in 2019, rel. change + 4.7%). A continuous increase was observed between 2005 and 2018 (14,878 [87.2%] followed by a slight decrease from 2018 until 2019. The rate of OR decreased until 2019 (48.1% in 2005 to 15.7% in 2019, rel. change -67.4%), the rate of EVAR increased (19.5% in 2005 to 50.2% in 2019, rel. change + 157.7%). The rate of hospitalizations with combined surgical treatment and EVAR increased from 0.4% to 4.9% (rel. change + 1,050.3%). In-hospital mortality (-41.4%) as well as in-hospital stay (-41.7%) decreased until 2019 (Table 1, Fig. 1C).

An 11.7% decrease in the absolute number of hospitalizations was observed in 2020 compared to 2019 (13,887 [86.5%] to 12,098 [85.3%]) while the rate of OR increased by 4.7% (15.7% in 2019 and 16.4% in 2020). During 2021, a slight further decrease was reported compared to 2020 (11,278 [85.0%]). (Table 2). During the first wave of the SARS-CoV-2 pandemic in spring 2020 we observed a relative decline of hospitalizations of 3.4%. No rebound was recorded in the summer plateau 2020 (0.0%), but this was followed by a further drop during the second wave of infection in late autumn of 1.2%, the third wave (-1.4%) and the fourth wave (-0.6%). Also during the summer plateau 2021 a decrease in hospitalizations was shown (-2.9%). During all waves of infection, a slight increase in EVAR was observed, whereas a slight increase in OR was reported between the waves of infection (Table 3, Fig. 2).

## Discussion

This study reports German nation-wide long-term data of hospitalizations due to ruptured and non-ruptured AAA between 2005 and 2021. While the number of ICD classified nrAAA steadily increased until 2015 and remained stable until 2019, the number of rAAA constantly decreased. The rate of OR and in-hospital mortality decreased over time, while the rate of EVAR and combined treatment increased from 2005 to 2019. During the SARS-CoV-2-pandemic we observed an increasing rate of in-hospital mortality and a decrease in hospitalizations due to nrAAA with a relative increase in the rate of rAAA, especially during the waves of infection.

The increase in hospitalizations of patients with nrAAA and decrease in hospitalizations of patients with rAAA by 2019 are consistent with a previous study reporting German nationwide healthcare data [2]. Similar results were reported in other countries, e.g. Sweden and Norway [5, 9–11]. In our study, the highest number of hospitalizations per year was observed in 2018, followed by a slight decrease in numbers of hospitalizations for nrAAA between 2018 and 2019. These findings are also consistent with previously reported data from Sweden [5]. The increase in hospitalizations for nrAAA may be explained by the introduction of screening programs, the most recent one in 2018 for men aged over 65 years [12]. However, this effect is possibly levelled out by the declining prevalence of AAA in recent years with stable hospitalizations between 2010 and 2017 and a slight decrease in 2019 [5].

In-hospital mortality constantly decreased between 2005 and 2019 (-34.0%), with nrAAA experiencing a greater decrease than rAAA (-41.4% vs -12.7%). Similar results have been reported in previous studies [2, 5, 9, 10, 13] and could be explained by earlier detection of AAA in screening programs and improvements of treatment methods.

As previously reported, the EVAR rate steadily increased while the OR rate decreased through 2019 [2, 5]. These findings are conforming with the European Society of Cardiology (ESC) guideline published in 2014 that recommended EVAR for patients with appropriate anatomy [14].

The data reported in our study showed a slight relative decrease of EVAR and a relative increase of OR from 2018 to 2019, which might be due to the publication of the recommendation by the National Institute for Health and Care Excellence (NICE) in the UK in 2018 [15, 16]. It clearly stated that patients with nrAAA should not be offered EVAR if OR was possible. These recommendations classified EVAR as an alternative equivalent to OR only in patients with rAAA. In contrast, the European Society of Vascular Surgery in 2019 took a more moderate view in its comprehensive management guidelines for AAA treatment based on a much broader literature base [17]. They recommended that EVAR should be used as the preferred treatment modality in patients with a reasonable life expectancy of about 2 to 10 years and suitable anatomy. The effect of this discussion on treatment decisions regarding nrAAA seem to have been subtle, but nevertheless, in 2019, a discreet but visible opposing trend was seen for the first time as described above.

Between 2019 and 2020, as well as between 2020 and 2021, we observed a decrease in the absolute number of hospitalizations due to nrAAA with a constant number of rAAA. One explanation may be a continuation of earlier trends in which a declining prevalence of AAA has been reported [5]. However, regarding specific periods during the waves of the SARS-CoV-2 pandemic, we reported a considerable decrease in AAA, especially during the first wave in spring (-25.5%) and a relative decrease in the rate of nrAAA (-3.4%). Therefore, it seems logical to assume that there is an association between the lockdown measures during the waves of infection and the low number of nrAAA hospitalizations. Other explanations could include patients fear of infection with SARS-CoV-2 during hospitalization and interruption of screening programs. Also during 2021, we observed a decline in hospitalizations for nrAAA, whereas no rebound was observed, not even between the waves of infection. These findings suggest that there is an accumulation of patients with untreated AAA that have potentially a higher risk of rupture in the next years and ought to receive special attention.

Interestingly, the decrease of nrAAA hospitalizations was continuously lower during the further waves of infection. These findings were similar to a tendency observed in a previous study that reported the impact of the SARS-CoV-2 pandemic on stroke treatment [18]. Possible explanations include guidance for patients to seek medical help in emergencies, introducing and communicating effective hygiene measures during the first wave of infection, and reducing thereby patients’ fear of infection [18, 19].

In-hospital mortality increased in 2020 compared to 2019 and in 2021 compared to 2020. These findings might be explained by the increasing rate of rAAA during both years, which are associated with higher in-hospital mortality. The data might also suggest hospitalization of patients with more advanced disease stages.

The total number of rAAA did not considerably decrease in 2020 and 2021 compared to 2019. However, in terms of the waves of infection, a relative increase of up to 22.9% (compared to the rate of rAAA in the same pre-pandemic time period) was reported. Interestingly, the rate of rAAA was also higher during the summer plateaus, especially in 2021 (+ 19.2%). Absolute numbers were, however, stable. Therefore, we cannot observe a rebound between the waves of infection.

The used healthcare data contained individual hospitalizations rather than individual patients and clinical information constrained to the ICD-10 catalogue. Especially in patients with rAAA, multiple hospitalizations per year were unlikely; however, bias could occur limiting the reported results. Furthermore, patients with rAAA, who did not reach the hospital, were not included and therefore, a potentially increased number of deaths due to rAAA during the pandemic could not be determined. According to the German health care system, all data were recorded to receive financial compensation. Therefore, a bias for economic reasons as well as errors in manual data entry cannot be ruled out.

## Conclusion

In recent decades, the number of hospitalizations for nrAAA and the rate of EVAR increased, whereas, as a result of efforts to improve the quality of care, in-hospital mortality, the number of rAAA and the OR rate decreased. In the first two years of the SARS-CoV-2 pandemic in Germany, the number of hospitalizations for nrAAA decreased, with an increasing rate of rAAA. These data draw attention to a potential deficiency in AAA treatment during the pandemic with a subsequent elevated risk of aneurysm ruptures. Awareness of the specific risk for certain patient groups should be improved and, if necessary, supported by the introduction of new screening initiatives.

### Supplementary Information

**Additional file 1: Supplemental Table 1**. Specific OPS-codes for surgical and endovascular treatment regimens.

**Additional file 2: Supplemental Table 2.** Hospitalizations due to ruptured and non-ruptured abdominal aortic aneurysm between 2005 and 2020.

## Data Availability

The datasets used and/or analysed during the current study are available from the corresponding author on reasonable request.

## Abbreviations

AAA: Abdominal aortic aneurysm
CHD: Chronic heart disease
CKD: Chronic kidney disease
DM-2: Diabetes mellitus type 2
DLP: Diseases of lipoprotein metabolism
ESC: European Society of Cardiology
EVAR: Endovascular aortic repair
HT: Arterial hypertension
ICD-10: International Classification of Diseases, Tenth Revision
IQR: Interquartile range
MREC: Medical Research and Ethics Committee
nrAAA: Non-ruptured abdominal aortic aneurysm
OPS: Specific Operating and Procedure codes
OR: Open repair
rAAA: Ruptured abdominal aortic aneurysm
RDC: Research data center
Sd: Standard deviation
