# Supplementary material for: German nation-wide in-patient treatment of abdominal aortic aneurysm—trends between 2005 and 2019 and impact of the SARS-CoV-2 pandemic
Source: CVIR Endovasc. 2023 Aug 29;6:44. doi: 10.1186/s42155-023-00389-4 (PMC10465413; doi:10.1186/s42155-023-00389-4)
Supplement: Supplementary file 1 — Additional file 1: Supplemental Table 1. Specific OPS-codes for surgical and endovascular treatment regimens. [file 42155_2023_389_MOESM1_ESM.docx]

| **Supplemental Table 1 Specific OPS-codes for surgical and endovascular treatment regimens** | |
| --- | --- |
| **Treatment** | **OPS-code** |
| *Surgery* | - 5-382.33 - 5-384.5 - 5-384.6 - 5-384.7 - 5-384.8 - 5-386.33 - 5-388.33 - 5-389.33 - 5-38c.04 - 5-38c.14 - 5-38c.24 - 5-38d.04 - 5-38d.14 - 5-38e.04 - 5-38e.14 - 5-380.33 - 5-381.33 - 5-38f - 5-38g - 5-393.3 - 5-395.33 - 5-397.33 - 5-384a |
| *Endovascular treatment* | - 8-836.d4 - 8-836.e4 - 8-836.f4 - 8-836.g4 - 8-836.h4 - 8-836.j4 - 5-38a.1 - 5-38ac - 5-38au - 5-38av - 5-38aw - 5-38ax - 5-38ay - 8-840.04 - 8-840.14 - 8-840.24 - 8-840.34 - 8-840.44 - 8-840.54 - 8-841.04 - 8-841.14 - 8-841.24 - 8-841.34 - 8-841.44 - 8-841.54 - 8-842.04 - 8-842.14 - 8-842.24 - 8-842.34 - 8-842.44 - 8-842.54 - 8-843.04 - 8-843.14 - 8-843.24 - 8-843.34 - 8-843.44 - 8-843.54 - 8-849.04 - 8-849.14 |
| *Hybrid* | - 5-98a.0 |
