# Supplementary material for: German nation-wide in-patient treatment of abdominal aortic aneurysm—trends between 2005 and 2019 and impact of the SARS-CoV-2 pandemic
Source: CVIR Endovasc. 2023 Aug 29;6:44. doi: 10.1186/s42155-023-00389-4 (PMC10465413; doi:10.1186/s42155-023-00389-4)
Supplement: Supplementary file 2 — Additional file 2: Supplemental Table 2. Hospitalizations due to ruptured and non-ruptured abdominal aortic aneurysm between 2005 and 2020. [file 42155_2023_389_MOESM2_ESM.docx]

| **Supplemental Table 2 Hospitalizations due to ruptured and non-ruptured abdominal aortic aneurysm between 2005 and 2020** | | | | | | |
| --- | --- | --- | --- | --- | --- | --- |
| **Year** | **2005** | **2010** | **2011** | **2012** | **2013** | **2014** |
| **All hospitalizations, n**  Age, mean (± sd)  Men, n (%)  In-hospital death, n (%)  In-hospital stay, d, median (IQR)  **Comorbidities:**  -Primary HT  -DLP  -DM-2  -CKD  -CHD | 14,075  71.94 (±8.63)  12,058 (85.7%)  1,642 (11.7%)  11 (4-16)  7,900 (56.1%)  3,140 (22.3%)  1,619 (11.5%)  2,212 (15.7%)  4,967 (35.3%) | 15,819  72.79 (±8.83)  13,621 (86.1%)  1,497 (9.5%)  9 (5-14)  9,853 (62.3%)  4,929 (31.2%)  2,669 (16.9%)  3,491 (22.1%)  5,248 (33.2%) | 15,772  72.89 (±8,83)  13,562 (86.0%)  1,373 (8.7%)  8 (5-14)  11,444 (72.6%)  5,128 (32.5%)  2,715 (17.2%)  3,359 (21.3%)  5,197 (33.0%) | 15,962  73.09 (±8.88)  13,539 (84.8%)  1,342 (8.4%)  8 (5-13)  10,189 (63.8%)  5,462 (34.2%)  2,793 (17.5%)  3,515 (22.0%)  5,068 (31.8%) | 15,833  73.08 (±9.00)  13,485 (85.2%)  1,328 (8.4%)  8 (5-13)  10,165 (64.2%)  5,446 (34.4%)  2,823 (17.8%)  3,661 (23.1%)  5,006 (31.6%) | 16,049  73.15 (±8.96)  13,676 (85.2%)  1,278 (8.0%)  8 (4-12)  10,536 (65.6%)  5,787 (36.1%)  2,899 (18.1%)  3,749 (23.4%)  5,133 (32.0%) |
| **Ruptured abdominal aortic aneurysm**  Hospitalizations, n (%)  Age, mean (± sd)  Men, n (%)  **Treatment:**  -OR, n (%)  -EVAR, n (%)  -combined, n (%)  -no intervention, n (%)  In-hospital death, n (%)  In-hospital stay, d, median (IQR) | 2,449 (17.4%)  74.77 (±9.64)  1,942 (79.3%)  1,286 (52.5%)  92 (3.8%)  14 (0.6%)  1,057 (43.2%)  1,202 (49.1%)  5 (1-18) | 2,410 (15.2%)  75.16 (±10.19)  1,948 (80.8%)  1,176 (48.8%)  226 (9.4%)  33 (1.4%)  975 (40.5%)  1,118 (46.4%)  5 (1-17) | 2,203 (14.0%)  75.40 (±10.02)  1,758 (79.8%)  1,032 (46.8%)  242 (11.0%)  44 (2.0%)  885 (40.2%)  986 (44.8%)  6 (1-17) | 2,172 (13.6%)  75.73 (±9.78)  1,714 (78.9%)  1,004 (46.2%)  267 (12.3%)  60 (2.8%)  841 (38.7%)    989 (45.5%)  7 (1-18) | 2,209 (14.0%)  75.56 (±10.32)  1,772 (80.2%)  908 (41.1%)  332 (15.0%)  91 (4.1%)  878 (39.7%)  965 (43.7%)  6 (1-18) | 2,048 (12.8%)  75.63 (±9.93)  1,649 (80.5%)  824 (40.2%)  322 (15.7%)  90 (4.4%)  812 (39.6%)  892 (43.6%)  5 (1-16) |
| **Non-ruptured abdominal aortic aneurysm**  Hospitalizations, n  Age, mean (± sd)  Men, n (%)  **Treatment:**  -OR, n (%)  -EVAR, n (%)  -combined, n (%)  -no intervention, n (%)  In-hospital death, n (%)  In-hospital stay, d, median (IQR) | 11,626 (82.6%)  71.35 (±8.28)  10,116 (87.0%)  5,588 (48.1%)  2,264 (19.5%)  50 (0.4%)  3,724 (32.0%)  440 (3.8%)  12 (6-16) | 13,409 (84.8%)  72.36 (±8.49)  11,673 (87.1%)  3,997 (29.8%)  5,293 (39.5%)  481 (3.6%)  3,638 (27.1%)  379 (2.8%)  9 (6-14) | 13,569 (86.0%)  72.48 (±8.56)  11,804 (87.0%)  3,458 (25.5%)  5,784 (42.6%)  740 (5.5%)  3,587 (26.4%)  387 (2.9%)  9 (6-14) | 13,790 (86.4%)  72.68 (±8.66)  11,825 (85.8%)  3,059 (22.2%)  6,307 (45.7%)  788 (5.7%)  3,636 (26.4%)  353 (2.6%)  8 (5-13) | 13,624 (86.0%)  72.67 (±8.70)  11,713 (86.0%)  2,669 (19.6%)  6,366 (46.7%)  943 (6.9%)  3,646 (26.8%)  363 (2.7%)  8 (5-12) | 14,001 (87.2%)  72.79 (±8.75)  12,027 (85.9%)  2,726 (19.5%)  6,651 (47.5%)  966 (6.9%)  3,658 (26.1%)  386 (2.8%)  8 (5-12) |
| **Year** | **2015** | **2016** | **2017** | **2018** | **2019** |  |
| **All hospitalizations, n**  Age, mean (± sd)  Men, n (%)  In-hospital death, n (%)  In-hospital stay, d, median (IQR)  **Comorbidities:**  -Primary HT  -DLP  -DM-2  -CKD  -CHD  -COVID 19 | 16,385  73.25 (±9.06)  13,864 (84.6%)  1,252 (7.6%)  7 (4-12)  10,715 (65.4%)  6,115 (37.3%)  2,963 (18.1%)  3,948 (24.1%)  5,295 (32.3%) | 16,185  73.24 (±9.14)  13,740 (84.9%)  1,242 (7.7%)  7 (4-12)  10,809 (66.8%)  6,115 (37.8%)  2,899 (17.9%)  4,006 (24.8%)  5,309 (32.8%) | 16,313  73.38 (±9.04)  13,787 (84.5%)  1,244 (7.6%)  7 (4-12)  10,881 (66.7%)  6,343 (38.9%)  2,831 (17.4%)  4,148 (25.4%)  5,290 (32.4%) | 17,056  73.75 (±8.92)  14,568 (85.4%)  1,236 (7.2%)  7 (4-11)  11,511 (67.5%)  6,917 (40.6%)  2,943 (17.3%)  4,074 (23.9%)  5,450 (32.0%) | 16,051  73.81 (±9.04)  13,663 (85.1%)  1,235 (7.7%)  7 (4-11)  10,775 (67.1%)  6,553 (40.8%)  2,731 (17.0%)  3,861 (24.1%)  5,060 (31.5%) |  |
| **Ruptured abdominal aortic aneurysm**  Hospitalizations, n (%)  Age, mean (± sd)  Men, n (%)  **Treatment:**  -OR, n (%)  -EVAR, n (%)  -combined, n (%)  -no intervention, n (%)  In-hospital death, n (%)  In-hospital stay, d, median (IQR) | 2,180 (13.3%)  75.70 (±10.23)  1,723 (79.0%)  820 (37.6%)  395 (18.1%)  81 (3.7%)  884 (40.6%)  922 (42.3%)  6 (1-17) | 2,089 (12.9%)  75.66 (±10.31)  1,678 (80.3%)  740 (35.4%)  398 (19.1%)  88 (4.2%)  863 (41.3%)  923 (44.2%)  6 (1-16) | 2,243 (13.7%)  75.13 (±10.13)  1,812 (80.8%)  813 (36.2%)  442 (19.7%)  73 (3.3%)  915 (40.8%)  939 (41.9%)  7 (1-18) | 2,178 (12.8%)  75.98 (±10.26)  1,724 (79.2%)  754 (34.6%)  464 (21.3%)  83 (3.8%)  877 (40.3%)  924 (42.4%)  6 (1-15) | 2,164 (13.5%)  76.10 (±10.11)  1,743 (80.5%)  788 (36.4%)  455 (21.0%)  90 (4.2%)  831 (38.4%)  927 (42.8%)  6 (1-16) |  |
| **Non-ruptured abdominal aortic aneurysm**  Hospitalizations, n  Age, mean (± sd)  Men, n (%)  **Treatment:**  -OR, n (%)  -EVAR, n (%)  -combined, n (%)  -no intervention, n (%)  In-hospital death, n (%)  In-hospital stay, d, median (IQR) | 14,205 (86.7%)  72.87 (±8.81)  12,141 (85.5%)  2,385 (16.8%)  6,840 (48.2%)  1,079 (7.6%)  3,901 (27.5%)  330 (2.3%)  7 (5-12) | 14,096 (87.1%)  72.88 (±8.90)  12,062 (85.6%)  2,237 (15.9%)  6,780 (48.1%)  1,197 (8.5%)  3,882 (27.5%)  319 (2.3%)  7 (5-11) | 14,070 (86.3%)  73.10 (±8.82)  11,975 (85.1%)  2,131 (15.1%)  7,202 (51.2%)  695 (4.9%)  4,042 (28.7%)  305 (2.2%)  7 (5-11) | 14,878 (87.2%)  73.42 (±8.65)  12,844 (86.3%)  2,310 (15.5%)  7,846 (52.7%)  690 (4.6%)  4,032 (27.1%)  312 (2.1%)  7 (5-11) | 13,887 (86.5%)  73.45 (±8.81)  11,920 (85.8%)  2,176 (15.7%)  6,970 (50.2%)  678 (4.9%)  4,054 (29.2%)  308 (2.2%)  7 (4-10) |  |
